# Assessing potential desflurane-induced neurotoxicity using nonhuman primate neural stem cell models

**DOI:** 10.3389/ebm.2025.10606

**Published:** 2025-06-13

**Authors:** Cheng Wang, Leah E. Latham, Shuliang Liu, John Talpos, Tucker A. Patterson, Joseph P. Hanig, Fang Liu

**Affiliations:** ^1^ Division of Neurotoxicology, National Center for Toxicological Research/FDA, Jefferson, AR, United States; ^2^ Office of Center Director, National Center for Toxicological Research/Food and Drug Administration (FDA), Jefferson, AR, United States; ^3^ Office of Pharmaceutical Quality, Center for Drug Evaluation and Research/FDA, Silver Spring, MD, United States

**Keywords:** anesthetics, desflurane,, developing neurons, neural differentiation, neurotoxicity

## Abstract

Safety concerns about general anesthetics (GA), such as desflurane (a commonly used gaseous anesthetic agent), arose from studies documenting neural cell death and behavioral changes after early-life exposure to anesthetics and compounds with related modes of action. Neural stem cells (NSCs) can recapitulate most critical events during central nervous system (CNS) development *in vivo* and, therefore, represent a valuable *in vitro* model for evaluating potential desflurane-induced developmental neurotoxicity. In this study, NSCs harvested from the hippocampus of a gestational day 80 monkey brain were applied to explore the temporal relationships between desflurane exposures and neural stem cell health, proliferation, differentiation, and viability. At clinically relevant doses (5.7%), desflurane exposure did not result in significant changes in NSC viability [lactate dehydrogenase (LDH) release] and NSC proliferation profile/rate by Cell Cycle Assay, in both short term (3 h) and prolonged (24 h) exposure groups. However, when monkey NSCs were guided to differentiate into neural cells (including neurons, astrocytes, and oligodendrocytes), and then exposed to desflurane (5.7%), no significant changes were detected in LDH release after a 3-h exposure, but a significant elevation in LDH release into the culture medium was observed after a 24-h exposure. Desflurane (24 h)-induced neural damage was further supported by increased expression levels of multiple cytokines, e.g., G-CSF, IL-12, IL-9, IL-10, and TNF-α compared with the controls. Additionally, our immunocytochemistry and flow cytometry data demonstrated a remarkable attenuation of differentiated neurons as evidenced by significantly decreased numbers of polysialic acid neural cell adhesion molecule (PSA-NCAM)-positive cells in the desflurane-exposed (prolonged) cultures. Our data suggests that at the clinically relevant concentration, desflurane did not induce NSC damage/death, but impaired the differentiated neuronal cells after prolonged exposure. Collectively, PSA-NCAM could be essential for neuronal viability. Desflurane-induced neurotoxicity was primarily associated with the loss of differentiated neurons. Changes in the neuronal specific marker, PSA-NCAM, may help understand the underlying mechanisms associated with anesthetic-induced neuronal damage. These findings should be helpful/useful for the understanding of the diverse effects of desflurane exposure on the developing brain and could be used to optimize the usage of these agents in the pediatric setting.

## Introduction

While controversial, it is suspected that extended or multiple early-life exposures to general anesthetics may lead to later adverse cognitive development [1–8]. However, it remains uncertain if the neurodevelopmental deficits are caused by general anesthesia, the surgery/procedure used to treat the condition, or the condition itself [9, 10]. All approved general anesthetics including desflurane, have NMDA-type glutamate receptor-blocking or GABA_A_ receptor-enhancing properties. Desflurane belongs to the group of medicines known as general anesthetics. Inhaled desflurane is used to cause general anesthesia (loss of consciousness) before and during surgery in adults. It is also used as a maintenance anesthesia in adults and children after receiving other anesthetics before and during surgery. Available findings indicate that decreased excitation of NMDA receptors or increased activation of GABA_A_ receptors can induce wide-spread neurodegeneration in the developing rodent or monkey brain [11–14] and cause cognitive impairments [15–17]. Additionally, a population-based study showed that early exposure to anesthesia could be a significant risk factor for later learning disabilities [1]. Appropriate studies performed have not demonstrated pediatric-specific problems that would limit the usefulness of inhaled desflurane [18] in children after receiving other anesthetics. However, children 6 years of age and younger are more likely to have unwanted side effects, such as coughing, chest tightness, or trouble breathing, which may require caution in patients receiving this agent. Previous studies have demonstrated that exposure of the developing brain to gaseous anesthetics such as nitrous oxide plus isoflurane during the period of synaptogenesis produced exposure duration-dependent increases in neurotoxicity [4, 7, 8, 12, 19] and lasting behavioral changes [6, 16]. A growing body of data indicates that prolonged bouts of anesthesia in the developing brain may lead to neurodegeneration. It is proposed that anesthetic-induced neurotoxicity depends upon the concentration of drug used, the duration of exposure, and the age of neurodevelopment at the time of exposure [12].

Most nonclinical models of neurotoxicity rely on animals, however, some *in vitro* models [11, 13, 20, 21] have emerged that can provide similar data. Particularly, NSC models [14] with their capacity to reproduce the most critical developmental processes including proliferation and differentiation, may serve as an effective alternative when evaluating anesthesia-related neurotoxicity. Since nonhuman primates have a central nervous system comparable to humans relative to anatomy, physiology and development, monkey NSCs [21, 22] can reach a high degree of concordance. Thus, the utilization of highly relevant *in vitro* monkey NSC models, might serve as a “bridging” system to evaluate the cellular and molecular changes after anesthetic exposure. Translational observations could also be made by exploring issues related to pediatric desflurane exposure from nonhuman primate neural stem cells, thereby providing valuable information on the ability to better interrogate specific mechanisms and the ability to do large scale of science research that cannot easily occur *in vivo*.

The neural cell adhesion molecule (NCAM) serves as a temporally and spatially regulated modulator of a variety of cell-cell interactions. It is known that cells expressing polysialylated isoforms of NCAM (PSA-NCAM) have a markedly increased capacity for structural plasticity [23–25], and the polysialic acid modification of the neural cell adhesion molecule is involved in spatial learning and hippocampal long-term potentiation [26]. Meanwhile, as a neuronal specific marker, PSA-NCAM has been used to identify/define developing neurons [21, 25, 27].

For this study, it was hypothesized that: 1) NSCs from nonhuman primates (NHP) could recapitulate key findings from *in vivo* work that support “clinical-like” studies in the NHP; 2) desflurane-induced neural damage, if any, most likely depends on the duration of exposure; 3) desflurane-induced neural damage/neuronal-loss could be evaluated using biological assays and monitoring PSA-NCAM expression levels; and 4) measuring PSA-NCAM levels could serve as a key step (as a target molecule) to dissect the underlying mechanisms associated with anesthetic-induced neurotoxicity.

## Materials and methods

### Test agents

Desflurane was purchased from NexAir, LLC (Memphis, TN). Desflurane (5.7%) was driven by the delivery gas (mixed gas) of 21% oxygen, 5% CO_2_, and balanced by nitrogen.

### Cultures

Media for NSC proliferation (named “growth medium”) and for NSC differentiation (named “differentiation medium”) were purchased from VESTA Biotherapeutics (Branford, CT). Monkey NSCs were harvested from the gestational day 80 fetal monkey [28]. The cells were seeded on poly-D-lysine/laminin-coated dishes. Monkey NSCs were cultured in NSC growth medium [Vesta Biotherapeutics; Branford, CT (changed every 48 h)] until the cells reached confluence. The cells were then transferred to 35 mm Petri dishes (Corning Incorporated; Corning, NY) with round cover slips at a seeding density of 6 × 10^3^ cells/cm^2^, and 24 h after seeding the cells were used for NSC characterization, and/or were directed to differentiate using neural differentiation medium (Vesta Biotherapeutics; Branford, CT) for another 5 days, with culture medium changed every other day. The cells were cultured in a standard culture incubator with humidified air and 5% carbon dioxide at 37°C. The concentrations of oxygen and carbon dioxide in the chamber were continuously monitored.

To expose to desflurane, the cells/cultures were loaded into a VitroCell System (VITROCELL Systems GmbH; Waldkirch, Germany) where the concentrations of desflurane, oxygen, and carbon dioxide (CO_2_) were accurately maintained during the experiment. Control cultures were also loaded to the same system without exposure to desflurane.

### 5-ethynyl-2′-deoxyruidine (EdU) incorporation assay

The NSC proliferation rate was measured using an EdU staining kit [Click-iT^®^ EdU Alexa Fluor^®^ High-throughput Imaging (HCS) Assay, Invitrogen; Carlsbad, CA] as previously described [13, 14].

### Cell cycle

NSCs were detached and washed twice in cold PBS and centrifuged at 200 *g* for 5 min at 4°C. Cells were fixed in 70% ethanol for 1 h on ice. Cells were washed and treated with 0.25 mg/mL RNase (Qiagen) for 1 h at 37°C. Cells were then immediately stained with 10 μg/mL propidium iodide (PI; Millipore; Burlington, MA) for 30 min on ice. Samples were run on a BD LSR Fortessa flow cytometer and at least 100,000 events were captured. Cells were analyzed for PI positivity using FCS Express version 6 (*De Novo* Software).

### Assessment of neurotoxicity

#### Lactate dehydrogenase (LDH) release

The release of LDH into the culture medium occurs with loss of plasma membrane integrity, a process most often associated with acute cell death. The LDH (Roche Applied Science; Indianapolis, IN) release assay was performed as previously reported [20, 29] to determine cytotoxicity after desflurane exposure.

### Cytokine immunoassay

Cell lysate was collected from NSCs after exposure to desflurane or delivery of mixed air (control) using the Bio-Plex Cell Lysis kit (Bio-Rad; Kansas City MO) and protein concentration was measured by the DC Protein Assay kit (Bio-Rad; Kansas City MO). Cell lysates were then probed with cytokines and growth factors using the Bio-Plex Pro Human Cytokine 27-plex assay (M500KCAF0Y; Bio-Rad; Kansas City MO) according to the manufacturer’s instructions. During the assay, antibody specifically directed against the cytokine of interest was coupled to a color-coded bead and allowed to incubate with the sample. Then a biotinylated detection antibody was added creating a sandwich of antibodies around the cytokine. This mixture was then detected by streptavidin-PE. When each reaction was run through the bio-plex system the fluorescent intensity was measured and calculated by the Bio-Plex 200 (Bio-Rad; Kansas City MO) software using a standard curve, and data were analyzed in Bio-Plex Data Pro software.

### Immunocytochemistry and nuclear staining

Immunocytochemical staining was performed as previously described [21]. Specifically, a mouse monoclonal antibody to nestin (1:100 dilution in PBS/0.5% BSA/0.03% Triton X-100 solution, Millipore; Burlington, MA) was used to label neural stem cells; a mouse monoclonal antibody to polysialic acid neural cell adhesion molecule (PSA-NCAM; 1:500; Miltenyi Biotec Inc; Auburn, CA); and polyclonal antibody to GFAP (1:200, Millipore; Burlington, MA) were used to identify glial cells. Briefly, the cells were rinsed with phosphate-buffered saline (PBS), fixed with ice-cold 4% paraformaldehyde in PBS and permeabilized with 0.5% bovine serum albumin (BSA)/Triton X-100 in PBS for 1 h. The cells were incubated with primary antibody at 4°C overnight. Bound antibodies were revealed with FITC-conjugated sheep anti-mouse IgG second antibody, or rhodamine-conjugated sheep anti-rabbit IgG secondary antibody. DAPI, a nuclear stain dye, in the mounting medium was applied to determine total cell counts in the cultures. Cells were viewed using an Olympus IX71 microscope (Olympus; Center Valley, PA).

### Flow cytometry analysis of PSA-NCAM staining

Cultured neural cells were dissociated from culture dishes using Accutase™ Cell Detachment Solution (BD Biosciences; San Jose, CA), washed with PBS, filtered through a 70-µm Falcon^®^ cell strainer (Life Sciences; Tewksbury, MA), fixed in BD Cytofix Fixation buffer (BD Biosciences; San Jose, CA) and permeabilized with BD Phosflow Perm Buffer III (BD Biosciences; San Jose, CA). Non-specific antibody binding was blocked using Human Fc Block (1:300) (catalog #564220, BD Biosciences; San Jose, CA) for 30 min at 4°C in cell staining buffer. Cells were washed and resuspended in PE conjugated PSA-NCAM (1:50) (Miltenyi Biotec Inc; Auburn, CA) for 1 h at room temperature. Samples were washed twice and immediately ran on a BD Bioscience LSR Fortessa flow cytometer. Data were analyzed using FCS Express version 6 (*De Novo* Software).

### Statistical analysis

Statistical analyses were performed, and graphs were produced using GraphPad Prism 9 (GraphPad Software Inc.; San Diego, CA). Data were analyzed with GraphPad Prism 9 using the unpaired t-test, and expressed as mean ± SD. A p-value less than 0.05 was considered statically significant. Each treatment condition was assessed at least in triplicate, and experiments were repeated three times independently.

## Results

### Characterization of NSCs

**FIGURE 1:**
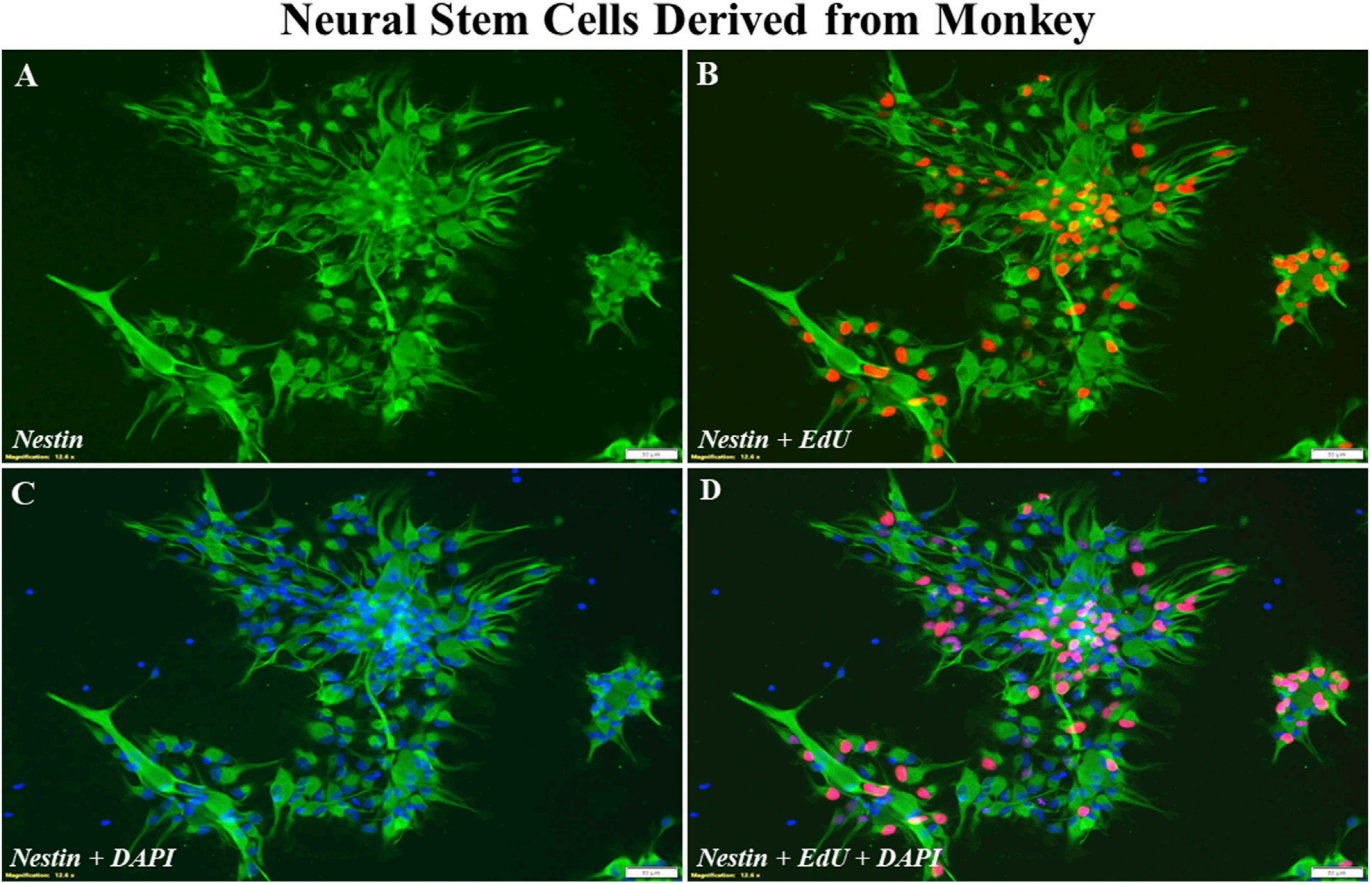
Representative photographs of immunostaining of nestin and EdU incorporation. In this control culture, numerous nestin positive NSCs [**(A)**; green] were EdU-positive [**(B)**; red]. These cells were undifferentiated NSCs when the cultures were maintained in the NSC growth medium. The total number of cells in the culture was revealed by DAPI-labeled nuclei **(C)**, and a merged image **(D)**. Approximately, on the day *in vitro* 8, the cells are confluent/ready for experiments. Scale bar = 50 µm.

**FIGURE 2:**
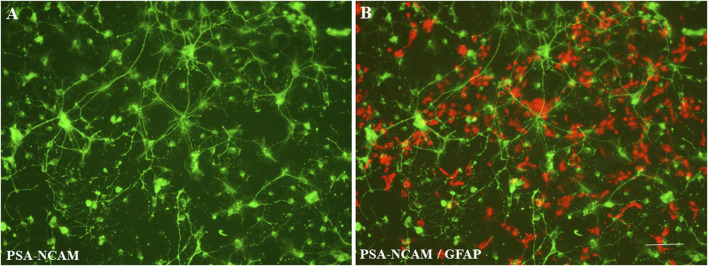
Representative photographs of NSC differentiation. Different neural cells (derived from monkey NSCs) with multiple processes and a clear neural network could be observed when the control cultures were maintained in neural differentiation medium. Morphologically defined neurons were positively stained by a monoclonal antibody to PSA-NCAM [**(A)**; green]. Typical astrocytes were labeled with the anti-GFAP antibody [**(B)**; red]. Scale bar = 50 µm.

The cultured monkey NSCs demonstrated the features of being able to self-renew (Figures 1A-C) and differentiate to generate lineages of neurons as well as glia including astrocytes and oligodendrocytes (Figure 2). As shown in Figure 1, numerous morphologically bipolar monkey NSCs were generated on day *in vitro* (DIV) 8, and most of these cells were positively stained by a NSC marker, nestin. In this control culture, the NSC proliferation was further confirmed using a commercially available EdU incorporation assay. The merged picture (Figure 1D; nestin/EdU/DAPI) shows that many of nestin-positive NSCs (green) were EdU-positive (red), demonstrating the capability of NSCs to proliferate.

Differentiated cells with multiple processes and a clear neural network were observed (Figure 2), when the cultures were maintained in neural differentiation medium for 5 days. Morphologically defined developing neurons were positively stained with specific neuronal marker, PSA-NCAM [A (green)], and typical astrocytes were labeled with the anti-GFAP antibody [B (red)].

### Cell cycle

**FIGURE 3:**
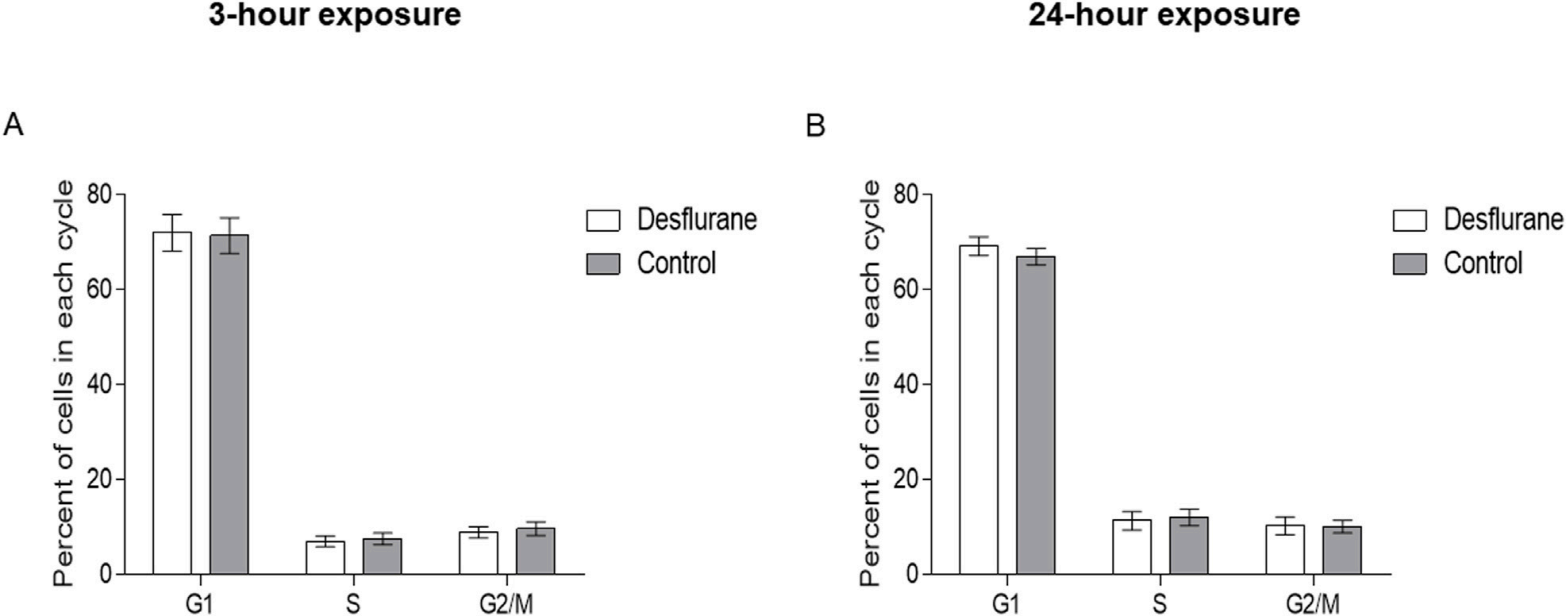
Proportion of cells in each phase of the cell cycle. Monkey NSCs were treated with 5.7% desflurane or delivery mixed air for 3 **(A)** or 24 **(B)** hours. Immediately following exposure, cells were collected and treated as described in the methods. Desflurane exposure does not significantly affect the proportion of NSCs in each phase of the cell cycle. Results are pooled from 3 independent experiments; a student’s T-test revealed no statistical significance.

Desflurane did not significantly affect the proportion of NSCs in each phase of the cell cycle, after 3-h (Figure 3A) or 24-h (Figure 3B) exposures, compared with the controls. There was no difference in the number of cells in S phase, indicating desflurane did not alter NSC differentiation. Results are pooled from three independent experiments.

### LDH release from NSCs and the differentiated cells

**FIGURE 4:**
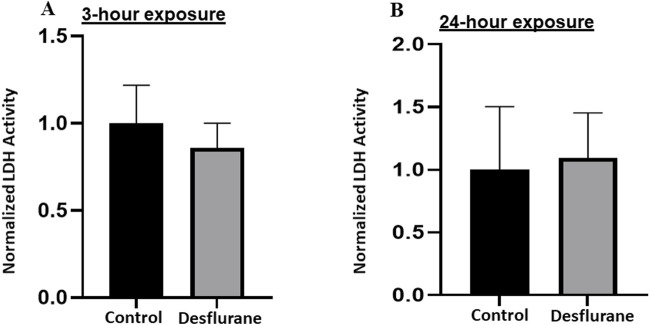
To study the vulnerability of NSCs to desflurane-induced neurotoxicity, LDH levels in the culture medium were monitored. No significant changes in the LDH release were detected when the monkey NSCs were exposed to desflurane (5.7%) in both short term (3 h) cultures **(A)** and prolonged (24 h) cultures **(B)**. The experiments were repeated three times independently.

At clinically relevant doses (5.7%), desflurane did not induce significant changes in LDH release, in both short (3 h) term (Figure 4A), and prolonged (24 h) exposure groups (Figure 4B).

**FIGURE 5:**
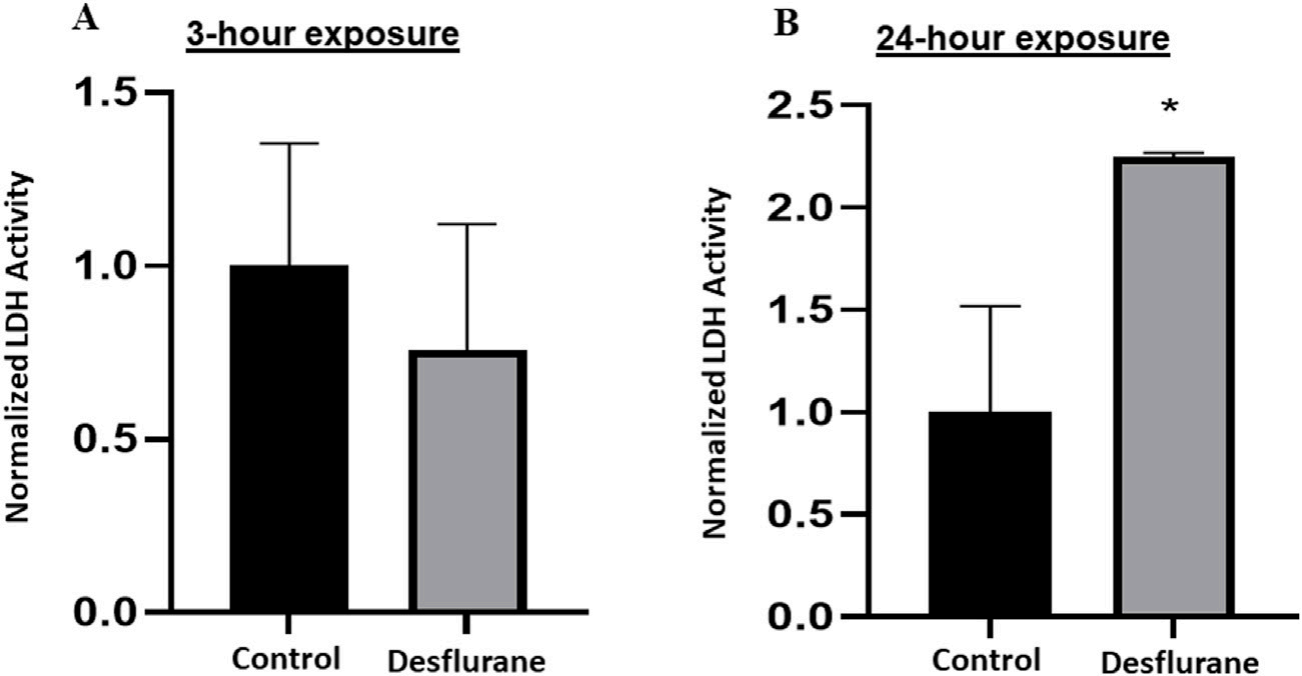
To study the vulnerability of differentiated neuronal cells to desflurane-induced neurotoxicity, LDH levels in the culture medium were monitored. Differentiated neuronal cell viability was not significantly affected after short term (3 h) desflurane exposure **(A)**. However, a significant elevation in LDH release into the culture medium was observed when these neuronal cells (derived/differentiated from monkey NSCs) were exposed to desflurane (5.7%) for 24 h **(B)**. The experiments were repeated three times independently. *p ≤ 0.05.

On the other hand, differentiated cells had a significant elevation in LDH release into the culture medium after 24-h exposure (Figure 5B), however, the 3-h exposure did not show a difference (Figure 5A). (Figure 5 about here)

### Cytokine changes in the differentiated cells

**FIGURE 6:**
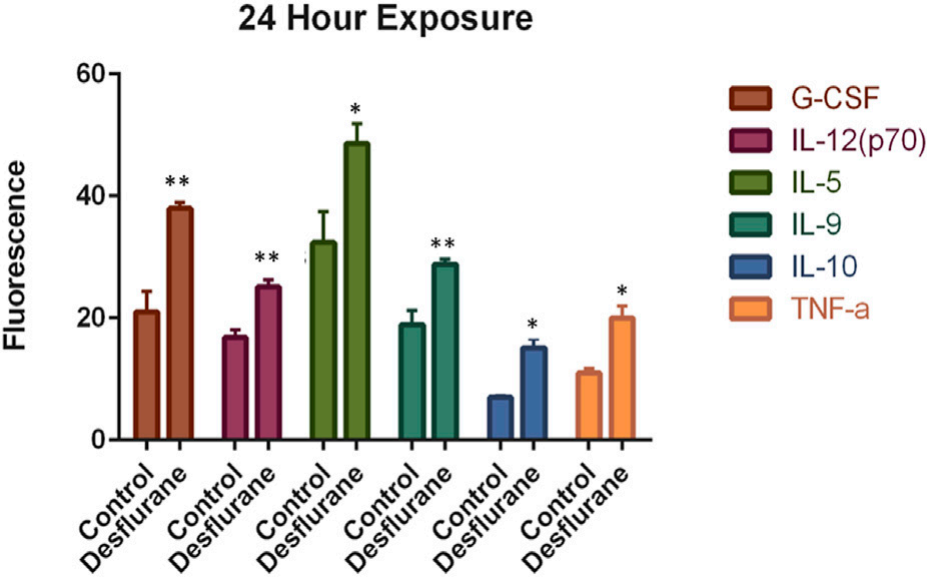
Desflurane induced inflammasome activation was evaluated using the Bio-Plex Pro Human Cytokine 27-plex assay. Prolonged desflurane (24 h) exposure resulted in significantly increased expression levels of multiple cytokines, including G-CSF, IL-12, IL-9, IL-10, and TNF- α , compared with the controls (delivery mixed air). Cytokine data were analyzed using unpaired T-test, and each experiment was repeated at least three times independently. *p ≤ 0.05, **p ≤ 0.01.

Desflurane exposure resulted in significant increases in levels of the key proinflammatory cytokines: interleukin 12 (IL-12) with an average 1.3-fold increase, G-CSF with an average 1.85-fold increase, TNF-α with an average 1.66-fold increase and anti-inflammatory cytokine such as interleukin 9 (IL-9) with an average 1.25-fold increase and interleukin 10 (IL-10) with an average 2.0-fold increase. These results (summarized in Table 1 and Figure 6) indicate that an enduring increase in systemic inflammatory cytokines may occur after 24-h desflurane exposure.

**TABLE 1:** Summary of Cytokines change in differentiated neural cells.

	P value	Mean1	Mean2	Difference	SE of difference	t ratio	df
Eotaxin	0.180113	14.3889	19.5833	–5.19444	3.20241	1.62204	4
FGF Basic	0.29853	18.9167	24.9722	–6.05556	5.0727	1.19376	4
G-CSF	0.00780072	21	38	–17	3.43929	4.94288	4
GM-CSF	0.67853	26.4444	30.1944	–3.75	8.40396	0.446218	4
IFN-g	0.141982	27.3611	39.3889	–12.0278	6.58908	1.82541	4
IL-10	0.538916	13.0278	14.6667	–1.63889	2.44208	0.671105	4
IL-12(p70)	0.00797281	16.7778	25.0556	–8.27777	1.68508	4.91238	4
IL-13	0.513043	11.9167	15.2222	–3.30555	4.61061	0.716945	4
IL-15	0.731475	26.9444	28.25	–1.30555	3.54708	0.368064	4
IL-17	0.510721	11.7222	15.5556	–3.83333	5.31565	0.721141	4
IL-1b	0.532207	9.22222	12.4444	–3.22222	4.71887	0.682837	4
IL-1ra	0.553413	8.86111	10.7778	–1.91667	2.96651	0.646102	4
IL-2	0.0763289	14.75	29.7222	–14.9722	6.3017	2.3759	4
IL-4	0.563121	8.63889	10.6944	–2.05556	3.26481	0.62961	4
IL-5	0.0532313	32.3889	48.6111	–16.2222	5.97384	2.71555	4
IL-6	0.510306	14.6389	17.3333	–2.69444	3.73247	0.721893	4
IL-7	0.943022	15	15.5833	–0.583332	7.66908	0.0760628	4
IL-8	0.922356	15.6389	15.8056	–0.166667	1.60631	0.103757	4
IL-9	0.015421	18.9444	28.8056	–9.86111	2.43226	4.0543	4
IL-10	0.004156	6.97222	15.1389	–8.16667	1.38666	5.88944	4
MCP-1(MCAF)	0.463354	18.8056	24.8333	–6.02777	7.44133	0.810039	4
MIP-1a	0.534612	8.44444	10.1667	–1.72222	2.53783	0.67862	4
MIP-1b	0.323724	20.1389	27.3611	–7.22222	6.42274	1.12448	4
PDGF-bb	0.745631	166.417	179.806	–13.3889	38.5134	0.347642	4
RANTES	0.063974	29.8056	39.3056	–9.5	3.74001	2.5401	4
TNF- α	0.0123884	10.9722	20	–9.02777	2.08685	4.32603	4
VEGF	0.213373	48	60.5556	–12.5556	8.49256	1.47842	4

**FIGURE 7:**
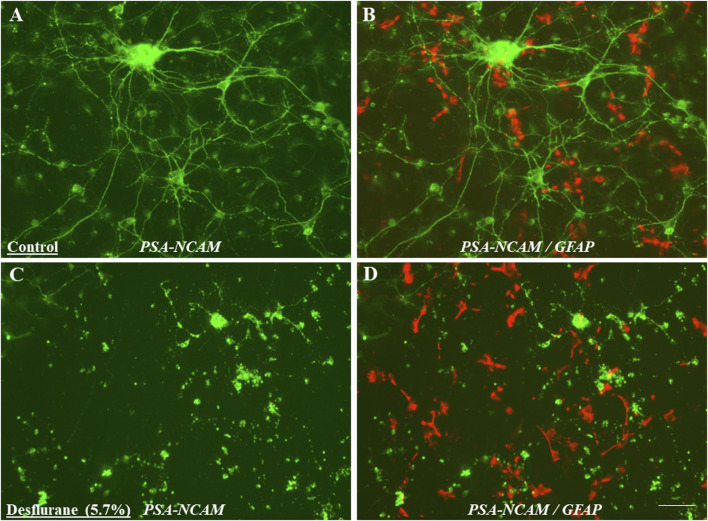
Differentiated neural cells with multiple processes and a clear neural network (cellular interactions facilitated through contact between cellular protrusions) could be observed after 5 days differentiation. Morphologically defined neurons were positively stained with monoclonal PSA-NCAM (neuron specific marker) antibody **(A)**. However, the number of PSA-NCAM positive neurons was obviously reduced in desflurane-exposed (prolonged) cultures. PSA-NCAM expression in desflurane group was exhibiting typically condensed residue pieces, fragmentation and shrinking profiles [**(C)**; green] compared with control [**(A)**; green]. In contrast, GFAP labeled astrocytes were not remarkably affected in the desflurane group [**(D)**; red], compared with control group [**(B)**; red].

Potential toxic effects after prolonged desflurane exposure (24 h) on differentiated neural cells were examined using immunocytochemical markers including neuronal and glial specific antibodies such as PSA-NCAM (neuronal specific marker), GFAP (astrocyte specific marker) and Galc [oligodendrocyte specific markers (data not shown)]. Numerous typical neurons were labeled by PSA-NCAM on their membrane surface of both cell bodies and processes in the control cultures (Figure 7A), and many differentiated astrocytes were labeled with the anti-GFAP antibody [Figure 7B (red)]. The number of PSA-NCAM positive neurons was obviously reduced after desflurane exposure. PSA-NCAM expression in the desflurane group exhibited typically condensed residue pieces, fragmentation and shrinking profiles (Figure 7C) compared with control (Figure 7A). In contrast, GFAP labeled astrocytes were not remarkably affected by desflurane (Figure 7D).

### Analysis of PSA-NCAM expression by flow cytometry

**FIGURE 8:**
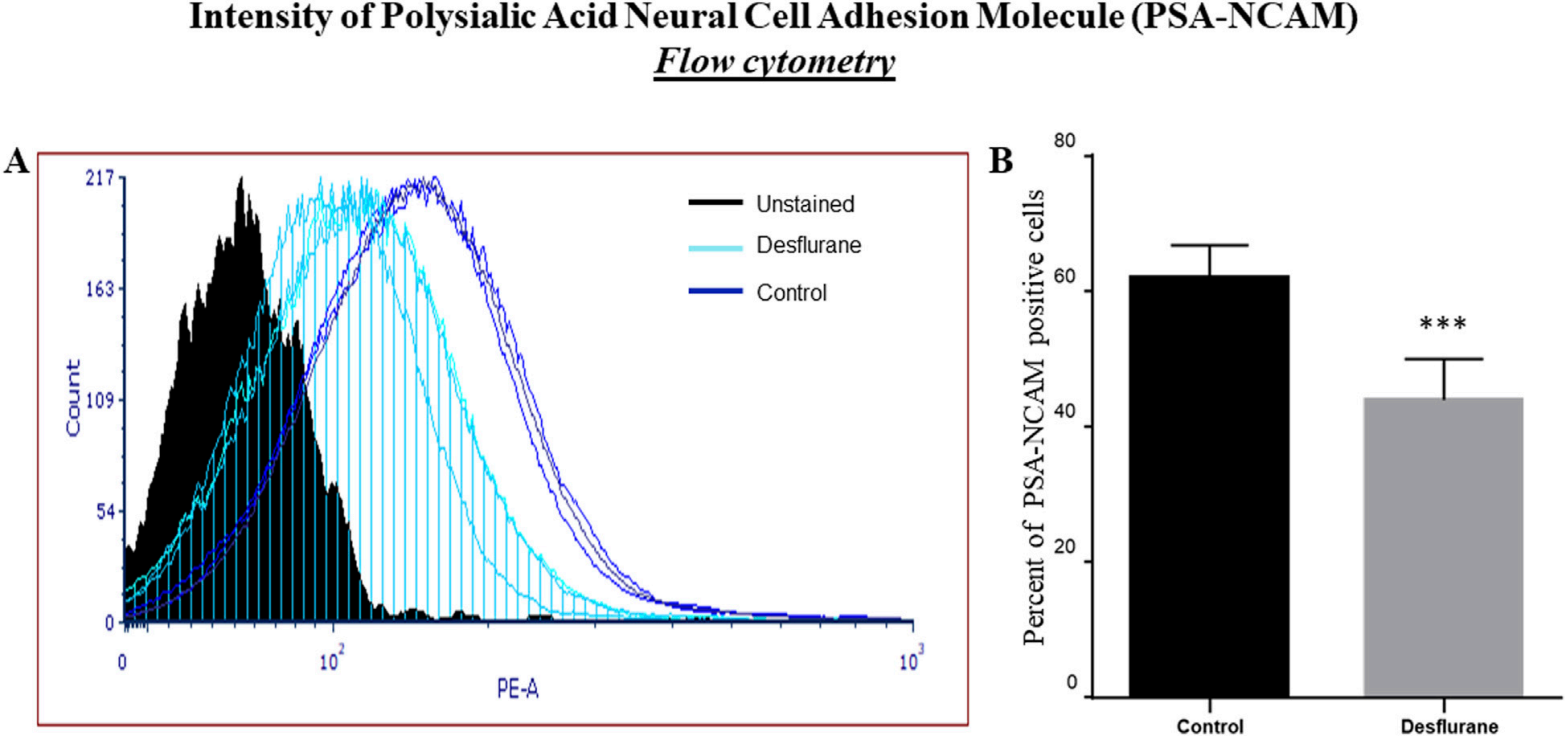
Representative flow cytometry histogram, normalized to mode, of PSA-NCAM staining. The black filled histogram is the unstained control. The light blue striped histograms are the desflurane exposed cells, and the solid dark blue histograms are the control air (delivery mixed air) exposed cells **(A)**. The percentage of single cells expressing PSA-NCAM after exposure to desflurane or control air is shown **(B)**. Our data indicated that prolonged (24 h) desflurane exposure at clinically relevant concentration specifically resulted in PSA-NCAM diminished expression. The data were pooled from three independent experiments. ***p < 0.0001.

Desflurane exposure diminished PSA-NCAM expression. Figure 8A shows a representative histogram. The black filled histogram is the unstained control. The light blue striped histograms are the desflurane exposed cells, and the solid dark blue histograms are the control air exposed cells. Figure 8B shows the percentage of single cells expressing PSA-NCAM after exposure to desflurane or control air. The data is pooled from three experiments. The experiments were repeated three times independently. Consistent with the immunocytochemistry data, the flow cytometry analysis (Figure 8) illustrates that 24-h desflurane exposure reduced the number of positively labeled PSA-NCAM cells, suggesting the reduction/damage of the developing neurons.

## Discussion

NSC culture is a key tool that can be used to assess potential anesthetic-induced neurotoxicity [13, 14]. Here, we used NSCs derived from fetal monkey multipotent stem cells to create a vitro model of the developing nervous system to study the pathophysiology of neurodegeneration associated with anesthetic (such as desflurane)-induced neurotoxicity [28]. Our data demonstrated that cultured monkey NSCs can proliferate on a dish and differentiate into neural cells (including neurons and glial cells). Our data highlights the effectiveness of the nonhuman primate NSC model to study anesthetic (e.g., desflurane)-induced neurotoxicity in the developing CNS.

Links between anesthesia exposure in developing brains and subsequent cognitive deficiencies have been identified in many pre-clinical studies [30–32]. Pre-clinically, both cell-culture and animal studies [11–14, 21] suggest that anesthetics may cause neural apoptosis, caspase activation, neurodegeneration, neuroinflammation, and ultimately, deficits in cognition. Desflurane is not often used for induction of anesthesia in children due to its potent airway irritant properties, which can lead to coughing, laryngospasm, and other complications. However, it can be used for maintaining anesthesia in children. Previously, an animal study in neonatal and adult mice demonstrated that desflurane exposure induced more neural apoptosis in neonatal mice compared to sevoflurane and isoflurane [33]. Further, adult mice exposed to desflurane demonstrated greater impairments in working memory compared to adult mice exposed to sevoflurane and isoflurane. It is important that any potentially deleterious side effects of anesthetics be elucidated and properly addressed. In the present study, the short-term or prolonged exposure of NSCs to a clinically relevant concentration of desflurane did not affect NSC proliferation and viability, suggesting NSCs are not sensitive to anesthetic-induced neurotoxicity. In contrast, the developing neurons were vulnerable to desflurane. The possible reason for the vulnerability may be due to the expression of GABA_A_ receptors on neurons. Our previous data demonstrated that no GABA_A_ receptor immuno-reactive staining was detected on nestin-positive NSCs (lacking physiological activation). However, strong immunoreactive staining for the GABA_A_ receptor was observed on neurons that were differentiated from NSCs [13]. Therefore, after differentiation, prolonged exposure to desflurane caused significant elevation of neuronal cell death and changes in cytokine levels, providing evidence that neuronal cells are a more vulnerable cell population than uncommitted NSCs to anesthetic-induced adverse effects in the developing nervous system.

Numerous preclinical animal studies suggest some evidence of neurotoxicity of volatile anesthetics [30, 34–36]. Moreover, numerous *in vivo* studies using a variety of models have demonstrated the potential of cognitive/developmental issues in children exposed to early-life anesthesia [35–37]. These concerns are balanced by a risk–benefit analysis. Currently, it is unknown if the *in vitro* models used here will have similar baseline and cell signaling (molecules) responses to desflurane exposure (short or prolonged) and alter developing neuronal transmission systems in corresponding *in vivo* models. Previously, neuroleptic anesthesia with a surgical insult (splenectomy) in rats resulted in signs of CNS inflammation and cognitive impairment on the first and third postoperative days, whereas control and anesthesia-only groups showed neither inflammatory changes nor behavioral effects [37]. Also, our previous *in vivo* studies [22] demonstrated that at a clinically relevant dosage, inhaled anesthetics (e.g., sevoflurane) induced and maintained a stable surgical anesthesia status in postnatal day 5 monkeys, and prolonged sevoflurane exposure resulted in significant alterations in gene expression profiles, cytokine secretion, lipid composition, and neuronal cell death. These data/findings are consistent with the view that prolonged exposure to inhaled agents could stimulate an inflammatory reaction in the CNS, which may contribute to cell death or other lasting negative consequences of early-life exposure to anesthesia.

Cytokines, key modulators of inflammation, are signaling molecules and are produced in response to invading pathogens [38–41]. It is well known that cytokines mediate neuronal and glial cell function to facilitate neuronal regeneration or neurodegeneration, and cytokine dysregulation is linked to microglial activation, neuroinflammation, neuronal damage, and cognitive deficits. In the present study, prolonged desflurane exposure of differentiated neural cells increased levels of multiple cytokines, including G-CSF, IL-12, IL-9, IL-10, and TNF-α, compared with controls. Neuroinflammation could play critical roles in the pathogenesis after longer durations of inhaled anesthetic exposure [22, 42]. Thus, altered cytokines could primarily be responsible for the development of increased neuroinflammation, and subsequent neuronal damage. Our data indicated that an enduring increase in systemic inflammatory cytokines (pro and anti-inflammatory cytokine accumulation) may occur after desflurane exposure. Elevated neurodegeneration after prolonged anesthetic exposure may involve an imbalance of pro- and anti-inflammatory cytokines [37, 42]. Since disturbed proinflammatory cytokines, especially TNF- α , have a fundamental role in modulating inflammation and could be responsible for a diverse range of signaling events within cells, leading to cell damage/death, it is tempting to speculate the potential of anti-proinflammatory drugs (and/or antibodies) to ameliorate/preclude anesthetic-induced neurotoxicity.

It has been reported that exposure to inhaled anesthetics produced transient changes in dendritic spine density in the developing brain [43], which may result in lasting changes to synaptic ultrastructure [44]. Longer durations of inhaled anesthetic exposure could inhibit LTP [45], and prolonged sevoflurane exposure decreased survival of neurons [46]. PSA-NCAM is a specific marker for developing neurons [21, 22]. Ultrastructural studies showed that during early development the polysialylated form of NCAM is expressed by growth cones, neuronal processes, and neuronal bodies [27, 47]. In the current study, substantial downregulation of PSA-NCAM expression was observed on the neuronal surface and their processes in prolonged desflurane-exposed neurons. In contrast, no significant differences including the number, size, form, and distribution of GFAP labeled astrocytes were observed between desflurane-exposed and control (mixed delivery air) cultures. Our flow cytometry analysis provided quantitative information on a reduced number of developing neurons and downregulation of PSA-NCAM expression levels with desflurane (24 h) exposure versus their control. These results highlight that differentiated neurons, not glia, are the most vulnerable cell population to prolonged desflurane-induced toxicity. In fact, PSA-NCAM is of protective interest/effect for its key role in promoting neuritogenesis and synaptic plasticity. Altered PSA-NCAM expression levels could result in significant changes in synaptic activity, synaptic formation, and synaptic remodeling [27, 48, 49], and consequently neuronal damage. Also, synaptic pruning and subsequent neuronal loss, whether by programmed cell death or necrosis, are critical to plasticity and stabilization of circuits in the developing nervous system and these are active processes that are tightly controlled by neurotrophins signaling mechanisms to ensure normal development and facilitate synaptic plasticity [50]. Previously, it was reported that PSA-NCAM interacts with NMDA-type glutamate receptors [51, 52]. PSA-NCAM could prevent glutamate-induced cell death [52] by restraining the signaling through GluN2B-containing NMDA receptors and regulating GluN2B-mediated Ca^2+^ influx in CA1 pyramidal cells in hippocampal slices [53]. It should be noted that PSA-NCAM may increase the sensitivity of neurons to brain derived neurotrophic factor (BDNF) and ciliary neurotrophic factor (CNTF) [54]. Also, PSA-NCAM could induce the activation of fibroblast growth fact receptor 1 (FGFR1) [55], and FGFR1/FGFs could critically impact *retinal ganglion cells* (*RGCs;* bridging neurons that connect the retinal input to the visual processing centers) survival [56]. Therefore, the dysregulated PSA-NCAM expression, after prolonged desflurane exposure, could suggest: 1) early neurodegeneration, 2) interruption of synaptic communication, 3) functional and/or behavioral deficits, 4) altered neuronal viability and plasticity, and 5) a target molecule in dissecting underlying mechanisms associated with prolonged anesthetic-induced neurotoxicity on uncommitted NSCs and differentiated neuronal cells. Collectively, the present study demonstrated that prolonged desflurane-induced downregulation/shedding of PSA-NCAM was closely related to the observed loss/damage of developing neurons.

It should also be mentioned that the VitroCell system used for toxicological testing of desflurane has some limitations. For example, 1) although temperature and humidity of the test have been conditioned and well controlled to meet specific requirements of the cell cultures, owing to the complexity of the physical processes governing desflurane transport and deposition, the submersed state of a cell culture is not exactly/absolutely representative of the situation in the respiratory tract. And 2) although each treatment condition was assayed at least in triplicate and the experiments were repeated three times independently, toxicological testing of desflurane requires more experimental replicates than three replicates which matters to ensure/facilitate the correct amount of drug was applied to each well and in a reasonably uniform/consistent manner and should be addressed in our future desflurane studies.

### Summary

Our findings suggest that at clinically relevant doses (5.7%) desflurane did not result in significant changes in NSC viability and NSC proliferation. In contrast, after differentiation, significantly elevated neuronal damage was detected after prolonged desflurane exposure. Our results indicated that an enduring increase in systemic inflammatory cytokines occurred after desflurane exposure, and the cytokine dysregulation could be a critical contributing factor to prolonged desflurane-induced neurotoxicity. In addition, the changes in PSA-NCAM expression confirmed the vulnerability of neurons in desflurane-induced neurotoxicity. Our experimental analyses provided quantitatively accurate and reproducible information regarding reduced neuronal viability and remarkable attenuation of PSA-NCAM levels after prolonged desflurane exposure.

### Future work

Continued pre-clinical investigation may have significant impact on desflurane’s clinical practice. Associated perturbations of the nervous system could be involved in blocking excitatory ion channels and increasing the activity of inhibitory ion channels. Since neural receptors/ion channels and PSA-NCAM expression levels, as well as calcium signaling, play crucial roles in receiving external signals and regulating intracellular signaling in numerous neurological processes, a study to monitor the baseline responsivity to neurotransmitters and changes in intracellular calcium concentrations would be informative for measuring states of cellular activity, neuronal viability and neuronal plasticity associated with anesthetic (desflurane)-induced neurotoxicity.

## Data Availability

The raw data supporting the conclusions of this article will be made available by the authors, without undue reservation.
